# ‘It's like these scientists own the rains’: indigenous knowledge, disaster warnings, and the politics of legitimacy in Malawi

**DOI:** 10.1111/disa.70028

**Published:** 2025-11-28

**Authors:** Stern Mwakalimi Kita

**Affiliations:** ^1^ Faculty of Resilience Rabdan Academy United Arab Emirates

**Keywords:** climate adaptation, disaster risk reduction, early warning systems, epistemic injustice, indigenous knowledge, knowledge pluralism, Sub‐Saharan Africa

## Abstract

This study examines the declining use of indigenous knowledge (IK) in early warning systems for climate‐related disasters in Malawi, drawing on qualitative data from four disaster‐prone districts and national‐level institutions. While IK is frequently referenced in policy discourse and programmatic frameworks, its practical integration into disaster risk reduction (DRR) efforts remains limited, underlining deeper epistemic tensions that structure disaster governance. The findings reveal that scientific systems are institutionally privileged owing to donor logics, technocratic norms, and standardised metrics, while IK is increasingly marginalised, both by formal structures and shifting community dynamics, including youth disengagement, intergenerational disconnects, and religious beliefs. Adopting a co‐productionist lens, the study argues for a move beyond tokenistic inclusion and towards genuine knowledge pluralism, recognising the distinct value of IK in fostering resilience, particularly in resource‐constrained and culturally diverse settings. The paper contributes to ongoing debates on epistemic justice, legitimacy, and the politics of knowledge in DRR and climate adaptation.

## 1 INTRODUCTION

In the face of escalating climate change impacts, low‐income countries such as Malawi, a landlocked nation in southeast Africa, are disproportionately vulnerable to climate‐related disasters (Ngongondo et al., 2021; Phalira et al., 2022). In contexts where formal infrastructure and scientific knowhow remain limited, many communities have relied historically on indigenous knowledge (IK) systems to anticipate and respond to environmental hazards. These systems, being locally embedded, orally transmitted, and socially validated, offer place‐based insights into climatic shifts, often through ecological signals such as flowering plants, animal migrations, or wind patterns (Shaw, Sharma, and Takeuchi, 2009; Mercer et al., 2010).

The relevance of IK has been widely acknowledged across fields such as forestry, fishing, and agriculture, including in relation to disaster risk reduction (DRR). For example, the Mamanwa people of the Philippines use bird behaviour to anticipate typhoons (Cuaton and Su, 2020), while in Malawi, communities associate the movement of hippopotamuses, snails, and ants with the onset of flooding (Trogrlić et al., 2019; Phalira et al., 2022). Scholars such as Iticha and Husen (2019) show how Borana pastoralists in Ethiopia read environmental cues to forecast drought. In this framing, IK is seen as a resilient, context‐specific knowledge base that complements scientific forecasts and enhances local preparedness (Trogrlić et al., 2021).

A growing body of literature highlights how the erosion of IK is taking place collectively owing to external pressures—such as modernisation, climate change, and environmental degradation—and deeper structural and epistemic forces. As Hadlos, Opdyke, and Hadigheh (2022) note, IK is often excluded from formal disaster management systems because of assumptions of inferiority, a lack of documentation, or perceived superstition. Yet, the diminishing use of IK is not simply the result of modernisation or environmental degradation; rather, it reflects what Fricker (2007, 2012, 2013) calls epistemic injustice: a process in which certain knowledge systems are systematically excluded from institutional decision‐making. Scientific systems, often externally imposed and treated as objective, are privileged within formal disaster governance frameworks, whereas IK is framed as anecdotal, superstitious, or irrational. This tension reflects broader dynamics of epistemic politics, where what counts as legitimate knowledge is shaped not only by evidence but also by institutional power, authority, and globalised development paradigms (Jasanoff, 2003, 2004; Leach, Scoones, and Wynne, 2005).

Despite growing calls for integration (Ziervogel and Opere, 2010; Masinde and Bagula, 2011; Makwara, 2013; Wang et al., 2019; Zulfadrim, Toyoda, and Kanegae, 2019; Pandey and Basnet, 2022), IK remains poorly documented, inconsistently validated, and underestimated in DRR planning. Generational shifts, ridicule, religious beliefs, and intra‐community contradictions all contribute to its fading use (Trogrlić et al., 2019). Simultaneously, scientific early warning systems, which are often under‐resourced and externally funded, struggle with coverage, accessibility, and sustainability. These tensions raise critical questions about whose knowledge counts, how legitimacy is assigned, and what inclusive disaster governance should look like.

Using a qualitative interpretivist approach, this study explores how IK is practised and interpreted in Malawi, what social and institutional forces contribute to its diminishing use, and how these dynamics reflect broader tensions in the politics of disaster‐related knowledge. Grounded in empirical fieldwork and informed by theories of co‐production, knowledge pluralism, and epistemic governance, the paper argues for more inclusive, justice‐oriented approaches to DRR that recognise and reconfigure the institutional architectures moulding knowledge legitimacy.

### 1.1 Conceptualising IK and its role in DRR

IK refers to locally embedded, context‐specific systems of understanding shaped by generations of interaction with the natural environment (Nakashima and Roué, 2002; Mercer et al., 2007). It encompasses beliefs, practices, values, and worldviews that are frequently transmitted orally and grounded in cultural, spiritual, and ecological relationships (Langill, 1999; Mutasa, 2015). Unlike scientific knowledge, which is typically generalised, quantified, and decontextualised, IK is qualitative, holistic, and experiential, based on close observation of environmental cues and social memory (Kelman, Mercer, and Gaillard, 2012; Iloka, 2016).

IK has been widely used in DRR to anticipate and respond to environmental hazards. Communities have employed IK to interpret early signs of floods, droughts, and storms through indicators such as animal behaviour, plant phenology, or atmospheric change (Iticha and Husen, 2019; Trogrlić et al., 2019; Cuaton and Su, 2020; Phalira et al., 2022; Mwalwimba, Manda, and Ngongondo, 2024).

The adaptive and community‐driven nature of IK has contributed to its continued relevance in DRR, especially in contexts with limited access to scientific infrastructure (Walshe and Nunn, 2012; Nkomwa et al., 2014). It is particularly valuable for localised forecasting and real‐time decision‐making, where scientific systems may be delayed or unavailable (Mercer et al., 2010; Trogrlić et al., 2021). IK is not monolithic, however: its transmission and application are influenced by social hierarchies, gender roles, and intergenerational dynamics (Kelman, Mercer, and Gaillard, 2012; Hadlos, Opdyke, and Hadigheh, 2022). Cuaton and Su (2020) highlight that men and women often hold distinct forms of ecological knowledge, fashioned by their differentiated experiences of resource use and social roles. In Malawi and elsewhere, younger generations may be less familiar with IK practices, leading to concerns about its erosion and loss (Mistry, 2009; Mutasa, 2015).

Despite its practical utility, IK continues to be undervalued in formal disaster governance frameworks. Efforts to integrate IK into scientific DRR systems have frequently sidelined its epistemological distinctiveness, reducing it to anecdotal evidence or folkloric data (Kamwendo and Kamwendo, 2014; Iloka, 2016). This marginalisation reflects broader structural and epistemic hierarchies, which are examined further in subsection 1.3.

### 1.2 Integration and its discontents: tensions in bridging knowledge systems

The idea of integrating IK into scientific systems in the field of DRR has gained attention in both academic and policy circles. Scholars argue that blending IK's localised, context‐sensitive insights with the predictive power and scalability of scientific forecasts can enhance the effectiveness of early warning, particularly in climate‐vulnerable settings (Mercer et al., 2010; Plotz, Chambers, and Finn, 2017; Pauli et al., 2021). IK systems are typically embedded in community practices and are perceived as more trusted and actionable at the grassroots level (Trogrlić et al., 2021), while scientific systems provide broader temporal and spatial data coverage.

Yet, practical efforts to integrate these knowledge systems often falter owing to fundamental differences in their epistemological foundations, institutional validation processes, and social legitimacy (Sillitoe, 1998; Nakashima and Roué, 2002; Hermans et al., 2022). Scientific forecasts tend to rely on numerical precision and standardised models, whereas IK is interpretive, qualitative, and deeply intertwined with cultural meanings (Kelman, Mercer, and Gaillard, 2012; Iticha and Husen, 2019). These ontological differences frequently lead to the dismissal of IK as ‘unreliable’, ‘unverifiable’, or ‘superstitious’ within formal disaster management systems (Hadlos, Opdyke, and Hadigheh, 2022; Hussein, Tole, and Mwakumanya, 2023).

In Malawi, for example, Phalira et al. (2022) and Ngongondo et al. (2021) have shown that IK is often regarded as inadequate for medium‐ to long‐range forecasting or cross‐boundary risks such as upstream floods. Some indicators, including animal behaviours or flowering patterns, are also becoming less visible due to environmental degradation and species decline (Trogrlić et al., 2019). But the limitations of IK are mirrored by those of scientific systems, which in many rural areas remain inaccessible, poorly localised, and overly dependent on donor funding (Kamwendo and Kamwendo, 2014; Ngongondo et al., 2021).

Moreover, integration efforts often fail to confront the unequal power relations that shape knowledge governance. Government agencies and non‐governmental organisations (NGOs) may selectively engage with IK only when it aligns with scientific data, while ignoring divergent interpretations or contested practices (Mapfumo, Mtambanengwe, and Chikowo, 2016). Institutional scepticism and a lack of political will to validate IK as legitimate knowledge further exacerbate its marginalisation (Dekens, 2007; Iloka, 2016). As Hermans et al. (2022) note, even participatory platforms can reproduce exclusionary dynamics if they do not explicitly address power asymmetries and validation politics.

Documentation of IK is frequently proposed as a solution to these challenges (Langill, 1999; Mutasa, 2015); however, critics contend that documentation can strip IK of its contextual nuance, detaching it from the sociocultural systems that give it meaning (Agrawal, 1995; Mistry, 2009). This risks transforming IK from a living practice into a static artifact, making it easier to control but less effective in action.

Ultimately, efforts to integrate knowledge systems without addressing the deeper epistemic hierarchies and institutional asymmetries risk reproducing the very exclusions they aim to overcome. As such, integration must be understood not just as a technical process, but also as a political and ethical project that requires more than harmonising tools. Instead, it demands deliberate shifts in whose knowledge is legitimised and how.

### 1.3 Epistemic politics and the legitimacy of knowledge

Discussions of IK in DRR inevitably intersect with deeper questions of epistemic politics; namely, how certain forms of knowledge are legitimised while others are dismissed, and what this reveals about power and authority in governance systems. The sidelining of IK is not merely a technical or capacity issue, but also part of broader struggles over who gets to define risk, act upon it, and shape policy responses. This dynamic resonates, as noted earlier, with what Fricker (2007) describes as epistemic injustice: the systematic exclusion of alternative knowledge systems from disaster decision‐making. While Fricker does not address IK specifically, her framework highlights how colonial legacies, developmentalist paradigms, and bureaucratic preferences for standardisation frequently undermine context‐specific and culturally grounded knowledge, particularly in low‐income countries like Malawi, where externally validated models often take precedence over locally embedded practices.

The theory of co‐production advanced by Jasanoff (2004) provides a useful lens here, emphasising that knowledge and social order are mutually constituted. What counts as ‘credible’ or ‘scientific’ is shaped not only by evidence, but also by institutional authority, funding structures, and the politics of expertise. Jasanoff (2003) contrasts ‘technologies of hubris’, which claim precision and control, with ‘technologies of humility’, which embrace uncertainty and inclusion. IK, when meaningfully engaged, embodies the latter. Yet, disaster governance systems often favour the former. Similarly, Leach, Scoones, and Wynne (2005) advocate for knowledge pluralism as a framework for engaging diverse knowledge systems in governance, a perspective that has been increasingly applied in DRR to support the inclusion of multiple epistemologies rather than forcing convergence around dominant paradigms.

In practice, however, disaster governance often reinforces hierarchies of knowledge. IK is frequently assessed on the terms of science, which is expected to produce standardised, quantifiable outputs, rather than being recognised for its interpretive, adaptive strengths (Sillitoe, 1998; Kelman, Mercer, and Gaillard, 2012). As a result, integration efforts may inadvertently relegate IK to a secondary or symbolic role: consulted but rarely incorporated into decision‐making processes. For instance, in Malawi, Phalira et al. (2022) found that while community members referenced more than 20 flood indicators, only two aligned with scientific data, raising not just concerns about local accuracy, but also deeper questions about whether scientific systems are even equipped to evaluate IK on its own terms.

These asymmetries are further reinforced by donor‐driven interventions and policy frameworks that valorise scientific technologies while overlooking or coopting community‐based systems (Agrawal, 1995; Hussein, Tole, and Mwakumanya, 2023). In some cases, well‐meaning efforts to document IK may inadvertently strip it of context and agency, transforming dynamic practices into static datasets that serve external agendas (Mistry, 2009; Mapfumo, Mtambanengwe, and Chikowo, 2016). This not only weakens the transmission of knowledge but also erodes community trust and ownership.

Critically, the legitimacy of knowledge is also influenced by internal community dynamics. Age, gender, and religious beliefs affect who is seen as a credible knowledge holder and what types of knowledge are retained or discarded (Mistry, 2009; Kelman, Mercer, and Gaillard, 2012; Mutasa, 2015; Sithole, Naser, and Guadagno, 2015; Cuaton and Su, 2020; Hadlos, Opdyke, and Hadigheh, 2022; Trogrlić et al., 2022). Youth may view traditional indicators as outdated or irrelevant, while religious institutions may discourage engagement with practices deemed ‘un‐Christian’ (Trogrlić et al., 2021). These intra‐community dynamics intersect with national and global trends, producing complex patterns of erosion, adaptation, and resistance.

By foregrounding epistemic politics, this study moves beyond simplistic narratives of knowledge loss or technical integration. Instead, it frames DRR as a site of epistemic governance, where competing claims to legitimacy are shaped by institutional power, donor agendas, and cultural authority. Drawing on Fricker's (2007, 2012, 2013) articulation of epistemic injustice, Jasanoff's (2003, 2004) co‐productionist lens, and Leach, Scoones, and Wynne's (2005) call for knowledge pluralism, the paper argues that meaningful integration of IK requires a fundamental rethink of whose knowledge is heard, how legitimacy is assigned, and what counts as credible evidence in disaster preparedness. The following section outlines the qualitative methodology used to examine how these dynamics unfold in the Malawian context.

## 2 APPROACH AND METHODOLOGY

This study adopts an interpretivist research paradigm to explore how IK systems are understood, practised, and contested in the realm of DRR and early warning in Malawi. Interpretivism is well‐suited to analysing socially constructed meanings, situated experiences, and the power‐laden nature of knowledge production, especially when working with epistemologies that have been marginalised historically (Saunders, Lewis, and Thornhill, 2019; Myers, 2020; Creswell and Creswell, 2022). Given the study's focus on the legitimacy, transmission, and integration of IK into disaster governance, a positivist or generalisable approach would have been ill‐equipped to capture the fluid, localised, and often contested realities surrounding it.

The interpretivist lens enabled the research to attend to the emic perspectives of community members and institutional actors, as well as to the cultural–symbolic dimensions through which risk, credibility, and warning are interpreted. Knowledge legitimacy is not merely a technical attribute but also a social and political claim, best accessed through deep qualitative engagement. A multi‐method qualitative approach, which combined key informant interviews (KIIs), focus‐group discussions (FGDs), observations, and embedded case studies, was therefore employed to capture the richness and variability of IK as it manifests across different regions, actors, and institutional contexts.

### 2.1 Research objectives

This study investigates how IK is used, interpreted, and contested in early warning systems for climate‐related disasters in Malawi, with a spotlight on floods and droughts. It aims to document IK practices and indicators across different communities, appraise how these are transmitted and valued, and assess the societal and institutional drivers of their diminishing use. In doing so, the research also explores the feasibility and implications of integrating IK into scientific systems. These empirical objectives are informed by a broader analytical concern with power, legitimacy, and the epistemic politics shaping disaster risk governance in low‐income settings.

### 2.2 Study location

**FIGURE 1:**
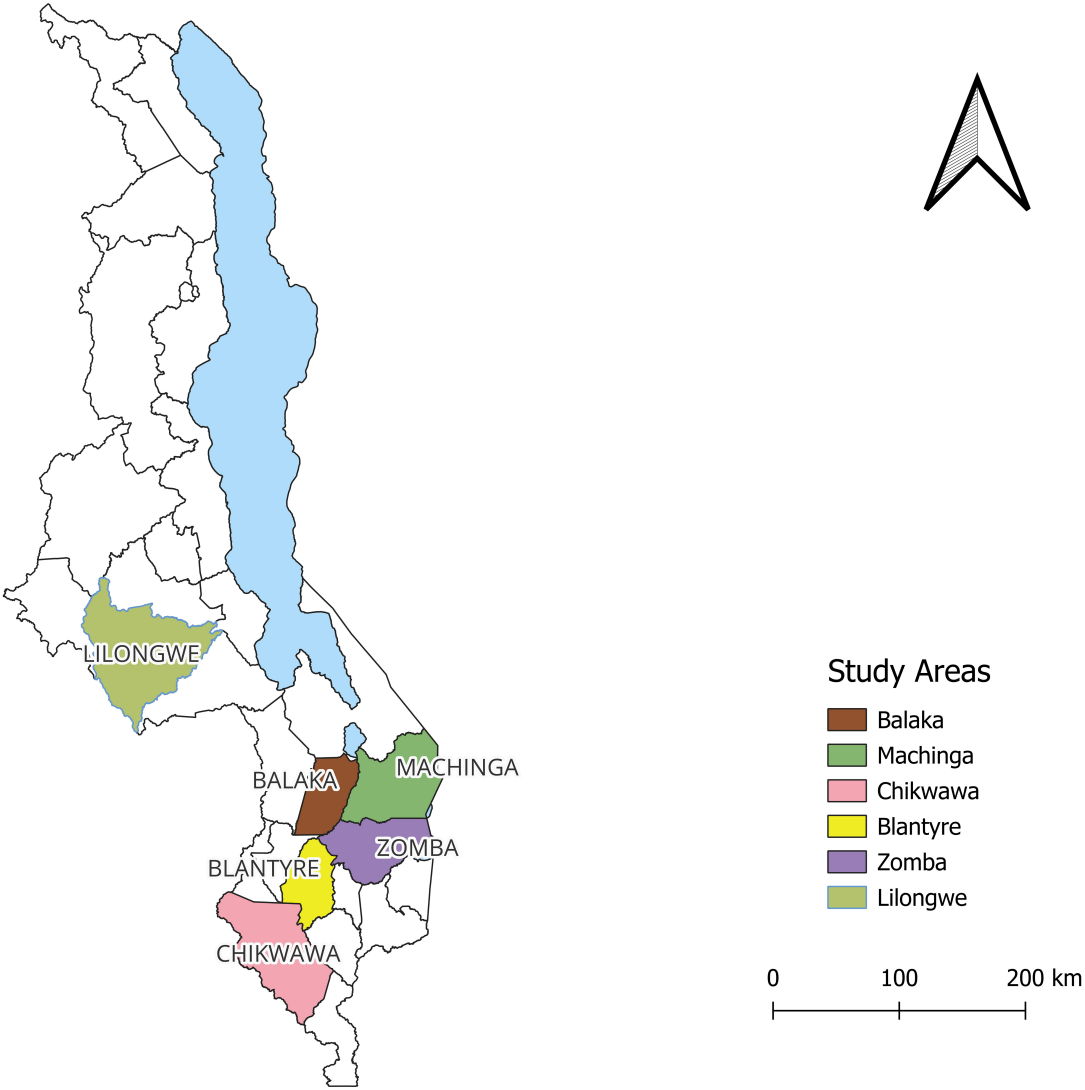
Map of Malawi showing the location of the study districts. **Source:** author, with support from Ms Jessie Phiri.

This study was conducted in 2020 across four disaster‐prone districts of southern Malawi—Chikwawa, Balaka, Machinga, and Zomba—with additional national‐level interviews held in Blantyre and Lilongwe (see Figure 1). These local and national data collection sites were purposively selected to ensure vertical coherence in the analysis of IK and early warning systems, combining community‐level experiences with national policy dynamics.

**FIGURE 2:**
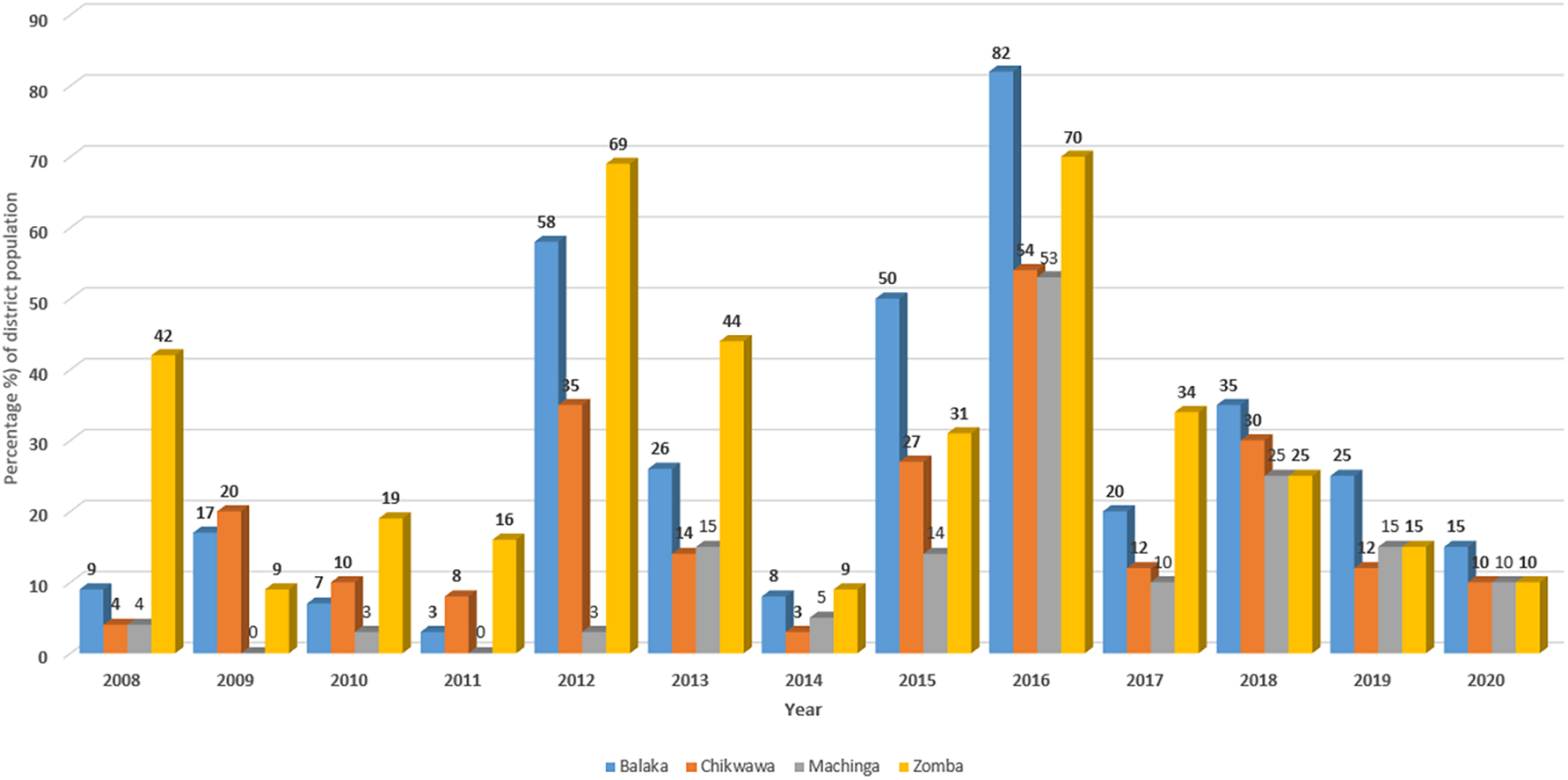
Percentage of population in study districts experiencing food insecurity, 2008–20. **Source:** author, based on various reports from 2008–20 by the Malawi Vulnerability Assessment Committee and the Integrated Food Security Phase Classification initiative.

The four rural districts were selected based on a combination of ecological diversity, hazard exposure, and sociocultural variation, which are factors that determine how IK is produced, transmitted, and valued in DRR. These districts are among the most affected by floods, droughts, and strong winds in Malawi, and have experienced recurrent disasters, including the devastating 2015 floods and Tropical Cyclone Idai in 2019 (Government of Malawi, 2019). Repeated shocks have disrupted rain‐fed agricultural systems and intensified food insecurity, trends reflected in Figure 2, which shows food insecurity prevalence from 2008–20.

Beyond hazard profiles, the sites differ in agro‐ecological zones, ethnolinguistic groups, and the institutional presence of NGOs and extension services. This diversity enabled the study to explore how IK functions across varying environments and governance arrangements, and how these factors affect its integration into formal early warning systems, or its marginalisation.

Incorporating Blantyre and Lilongwe added perspectives from government agencies, donors, and policy actors involved in national DRR and climate adaptation efforts. This multi‐scalar approach yielded a more holistic understanding of how disaster knowledge is governed, legitimised, or excluded across institutional levels.

### 2.3 Primary data collection

Primary data were gathered using qualitative methods including KIIs, FGDs, case narratives, and direct observation. These methods were chosen to explore the context‐specific, embedded nature of IK in DRR.

KIIs were conducted at the community, district, and national levels using semi‐structured guides focused on IK practices, perceptions of reliability, integration into scientific systems, and institutional support. Participants included lead farmers, local leaders, extension officers, civil protection members, and DRR officials. FGDs were held separately with elderly men, elderly women, youth, and persons with disabilities to capture diverse experiences. Group sizes were reduced (between three and six participants) to meet COVID‐19 (coronavirus disease 2019) safety protocols and to enhance open dialogue. Each session lasted between 60 and 90 minutes and was recorded with participant consent.

Case studies and observation were used to document IK indicators in situ, including plant, animal, and environmental signals. Snowball sampling helped to identify households with rich experiential knowledge. Informed consent was obtained on signed forms. Participants were assured of confidentiality and the right to withdraw at any point. Data were anonymised during transcription and analysis.

Data collection followed an open‐ended protocol that allowed respondents to describe locally‐known early warning signs without prompts or restriction. To ensure analytical clarity, only those indicators that were (i) described with reasonable consistency, (ii) locally recognised as predictive, and (iii) mentioned by multiple respondents were retained. Vague references, unnamed species, or inconsistently interpreted signs were excluded. This resulted in 241 unique indicators, categorised under animals, plants, hydrometeorological patterns, astrology, and others, as summarised in subsection 3.1.

### 2.4 Sampling and sample sizes

The study employed a purposive sampling strategy, selecting regions recently affected by climate‐related disasters to capture the lived experiences of those directly engaged in or impacted by early warning systems. This approach aligns with the interpretivist paradigm, which prioritises depth, contextuality, and meaning‐making over generalisability (Saunders, Lewis, and Thornhill, 2019; Creswell and Creswell, 2022).

Malawi's administrative structure begins at the village level and consists of households led by village heads. Multiple villages form a Group Village Head (GVH) area, and in turn comprise Traditional Authority (TA) areas. The study covered seven TAs across four districts, with Zomba including only one TA. At least one GVH area was selected per TA, and participants were identified with support from district officials and field staff familiar with local dynamics.

The sampling was designed to ensure demographic diversity, particularly in terms of gender, age, and vulnerability status. Participants included community leaders, youth, elderly individuals, and persons with disabilities, as well as district‐ and national‐level stakeholders such as government officials, NGO staff, and extension officers.

In total, the study engaged 80 key informants (30 females and 50 males) and 195 FGD participants (100 females and 95 males). These participants were selected based on their knowledge of or involvement in early warning systems and DRR activities. Separate sessions and interviews ensured that less dominant voices, especially women and youth, were heard.

FGDs were conducted with distinct demographic groups in each GVH, including male adults, female adults, male youth, and female youth. Of the 39 FGDs convened, 19 were with males and 20 were with females. Approximately 50 per cent of all FGDs involved youth, with groups comprising between six and eight participants. While youth participation in KIIs was more limited, around 30 per cent, this reflects the age profile of formal leadership, technical experts, and custodians of IK. Nevertheless, the inclusion of separate youth FGDs ensured that younger voices and perspectives on the evolution of IK systems were meaningfully captured.

To mitigate potential bias and enhance trustworthiness, participant recruitment emphasised community rapport and transparency. The lead researcher's shared linguistic and cultural background with most participants facilitated trust but also necessitated reflexive awareness of power dynamics and assumptions. These considerations were openly discussed during field debriefings to reduce interpretive bias in the data collection process.

### 2.5 Data analysis

Data were analysed using a grounded theory approach aligned with the study's interpretivist paradigm, which seeks to generate insights from lived experience rather than test predefined hypotheses (Glaser and Strauss, 1967; Heath and Cowley, 2004; Chametzky, 2016; Creswell and Creswell, 2022). This method was well‐suited to exploring the localised and under‐theorised nature of IK in disaster risk contexts.

Audio recordings and FGD transcripts were reviewed and coded using a constant comparative method. Two researchers independently developed and refined an initial codebook based on recurring patterns linked to the study's questions. Final themes were validated across the full dataset through collaborative discussion. Thematic clusters reflected both emic categories and broader concerns about integration, legitimacy, and transmission. The analysis considered variation across demographics and locations, including gendered knowledge and intergenerational shifts.

Reflexivity was maintained through field debriefings, with the lead researcher's cultural proximity enhancing access but requiring ongoing critical reflection. Triangulation of data sources (KIIs, FGDs, and observations) and participant types (community members, officials, and experts) supported the credibility of the findings. The key themes of trust, reliability, exclusion, and adaptation emerged inductively and were interpreted through the theoretical lenses of knowledge pluralism and epistemic politics, as discussed later in the paper.

## 3 RESULTS

### 3.1 Mapping the IK system: patterns, practices, and variability

The study documented 379 indigenous early warning indicators across the four disaster‐prone districts: Chikwawa (110); Balaka (109); Machinga (84); and Zomba (76). After removing duplicates within them, 241 unique indicators were retained.

Indicators were categorised in five groups: animal behaviour (39 per cent); plant‐based signs (29 per cent); hydrometeorological patterns (17 per cent); astrological signs (12 per cent); and other (3 per cent). Common examples included ant activity, owl hooting, and the toad grasshopper, all believed to signal rainfall patterns. Table 1 presents a district‐wise disaggregation of these 241 unique indicators. Animal‐based signs were consistently dominant across all four disaster‐prone districts, with Balaka recording the highest count (29). Plant‐based indicators were also widely cited, while astrological signs, such as lunar positioning or ancestral messages in dreams, were most commonly reported in Chikwawa and Balaka. Frequent cases cited were, inter alia, ant activity, owl hooting, and the toad grasshopper, all believed to signal rainfall patterns.

**TABLE 1:** District‐wise distribution of indigenous early warning indicators, disaggregated by source (n = 241).

District	Animal‐based	Plant‐based	Hydrometeorological‐related	Astrology‐related	Others	Total
Balaka	29	17	12	8	2	**68**
Machinga	22	14	13	3	1	**53**
Zomba	21	17	9	6	1	**54**
Chikwawa	23	22	7	12	2	**66**
**Total**	**95**	**70**	**41**	**29**	**6**	**241**

**Source:** author.

Many indicators were highly localised. The *njeza* (bead‐bean) plant, used to predict the onset of rain, was reported only in Zomba. Crocodile behaviour, python sounds, and specific bird species like Ng'ombeng'ombe were cited in select communities. An elder in Chikwawa commented: ‘When we heard the sound of these birds, we knew that it would rain within two days’.

Plant‐based signs, particularly tree flowering, were widely mentioned. Mango, baobab, and *nthundu* (broom‐cluster fig) trees were said to indicate rainfall quality and timing. One farmer noted: ‘To see the flowering of *nthundu* trees, we now have to go to the next village. It wasn't like this before’.

Meanings varied across locations, however. For example, mango flowering was seen as a sign of rain in one village and drought in another. Similar variation existed in interpreting *tsokonombwe* (toad grasshopper) sounds or *njeza* flowering: some communities linked them to rainfall, others to drought or flooding.

In some cases, participants could not identify specific plant or animal names, especially when describing behaviours or sounds. These ambiguities led to the exclusion of some indicators, such as indistinct bird calls or unnamed flowering trees.

Overall, the mapping revealed significant diversity and ecological specificity, with patterns shaped by geography, language, and community history.

### 3.2 Epistemic disruption: climate change, environmental degradation, and reliability erosion

Across all four disaster‐prone districts, participants reported that climate change and environmental degradation have disrupted the visibility, reliability, and interpretation of indigenous indicators. Deforestation, agricultural expansion, and habitat loss were frequently highlighted. A farmer in Chikwawa said: ‘There used to be this small bird we followed, but I haven't seen it in years’. An elder in Balaka added: ‘The crocodiles used to move closer to villages when heavy rains were coming, but now they're gone as rivers have dried up’.

Plant‐based signs have also diminished. A participant from Chikwawa stated: ‘We had this tree that would flower when rains were near. These days, you don't see it anymore. The land is cleared now’.

Erratic rainfall and shifting seasons have weakened indicator accuracy. ‘The rains used to come early in October, but now it's unpredictable’, said a leader in Machinga. Some signs have become inconsistent or misleading. A Chikwawa farmer remarked: ‘Even if there are a lot of ants that year, we don't get normal rains’.

Climate variability has also caused conflicting outcomes among indicators. One Zomba farmer enquired: “That mango tree is full of flowers, but others aren't. What should we conclude from this?’. The disappearance of indicator species and altered ecological behaviours were commonly linked to broader environmental change. An elder in Zomba noted: ‘There was a fish that swam upstream before heavy rains. Now, with the rivers drying from deforestation, we no longer see it’.

These disruptions have led to growing uncertainty, with many communities questioning IK's relevance and increasingly turning to scientific forecasts that are perceived as more consistent.

### 3.3 Erosion of practice: modernity, youth disengagement, and social norms

Participants described a marked decline in the intergenerational transmission and daily practice of IK, especially among youth. This erosion was linked to modern agricultural practices, shifting values, and the influence of religion and formal education.

Respondents observed that youth no longer engage with elders to learn traditional forecasting. An elderly participant in Zomba observed: ‘IK is mainly used by the old generation because this one is copying the Western culture… they no longer listen to us’. Even older respondents often described IK as *zamakolo*—that is, ‘of the ancestors’.

Literacy and modern extension services have reshaped farming practices. A Balaka farmer said: ‘We now follow what the extension workers tell us… the old ways are no longer reliable’. Others noted that radios, telephones, and schools have replaced traditional forums for knowledge sharing. One respondent explained: ‘Young people don't gather around the fire to hear stories or signs anymore; they have radios and phones now’.

Religious beliefs were also cited as barriers. ‘People say making sacrifices is unchristian, so we've stopped’, declared a participant in Zomba. Even when rituals continued, failed outcomes attracted ridicule. An elderly man in Chikwawa shared: ‘Last year, our leaders made sacrifices, but the rains didn't come. People laughed, so they stopped the practice’.

Some IK practices are now stigmatised or dismissed. Forecasting signs involving snakes or birds were sometimes mocked or linked to witchcraft. As a man in TA Chapananga, Chikwawa, put it: ‘Young people say that's a primitive way of thinking’. Across districts, generational shifts, religious norms, and education were consistently identified as key drivers of IK's decline.

### 3.4 Institutional marginalisation and ambivalence

Participants reported that government departments and NGOs rarely prioritise IK in early warning systems. While some stakeholders acknowledged its value, particularly in remote or resource‐limited areas, many described institutional preferences for scientific approaches. A lead farmer in Balaka emphasised: ‘Actually, the government is discouraging the use of IK. There is nothing they are doing against the threat to IK’.

Respondents noted that most institutional messaging encourages reliance on scientific systems. GVH Mangulu of Machinga shared: ‘We are mostly using the scientific ones because it is the one mostly encouraged by the government and different organisations’.

A few district‐level actors suggested coexistence was encouraged in principle. A local disaster risk management committee member in Balaka noted: ‘The government, through extension workers, is telling farmers not to completely ignore the traditional ways’.

In practice, however, participants described a lack of formal programmes to preserve or integrate IK. Extension efforts, training, and communication platforms were seen as overwhelmingly focused on scientific models.

Agricultural interventions by NGOs and government were also cited as unintentionally undermining IK. The introduction of hybrid crops altered phenological cues traditionally used for forecasting. A senior officer from the Department of Agricultural Extension Services stated: ‘NGOs and other farmers bring in hybrid or improved varieties of mango trees. So, you can't deduce from such varieties; that's a threat’. A participant in Zomba echoed the point: ‘These improved tree varieties flower early, but they don't follow the seasons. You can't use them to predict anything’.

Donor preferences further constrained the promotion of IK. Participants explained that IK‐related projects struggle to attract funding, as donors prioritise scientifically verifiable indicators. One development partner remarked: ‘Even when requesting support, you can't say people are seeing ants and need help. No donor will fund that’.

### 3.5 Contesting reliability: knowledge, trust, and the shifting value of IK

Many participants expressed declining trust in the reliability of indigenous early warning indicators. While IK was historically central to seasonal decision‐making, its predictive accuracy was perceived to have weakened. A participant from Chikwawa remarked: ‘I see the number of people using IK declining because it is not giving us what we expect. For example, you will see the birds or animals making noise just like before, but sometimes the rains don't come’.

This uncertainty was compounded by inconsistent interpretations across locations. In Zomba, one participant underlined: ‘People don't agree on what the same sign means anymore. One village says it's rain, the next one says it's drought. It confuses us’.

Discrepancies were common for indicators such as the *tsokonombwe* and *njeza* plant. Participants stressed that many indicators are closely tied to seasonal milestones, making them less adaptable to increasingly erratic patterns. An elder said: ‘The rains used to come early in October, but now it's unpredictable… and the signs we used to follow no longer work’.

In this shifting context, many participants expressed a growing preference for scientific forecasts, seen as more consistent and responsive. GVH Lundu of Chikwawa observed: ‘Usually, the scientific information comes to pass. It's like these scientists own the rain. Last year they told us exactly when the rains would start and stop. We planted early as advised, and it happened as predicted’.

Even so, scientific systems were not viewed as infallible. Some participants noted that forecasts also fail, and that both systems require cautious interpretation. One farmer pointed out: ‘Some plants may produce more flowers due to the amount of water they absorb, so we shouldn't automatically conclude that heavy rains are coming’.

### 3.6 Structural fragility and sustainability risks in scientific forecasting

While scientific early warning systems are increasingly trusted by communities owing to their geographic coverage and perceived accuracy, participants raised concerns about their long‐term sustainability. Many systems, especially those based on meteorological and hydrological monitoring, depend heavily on donor funding for installation, maintenance, and upgrades. This reliance creates structural vulnerabilities often overlooked in community narratives of trust. As one officer from the Department of Disaster Management Affairs stated: ‘Virtually every advanced scientific early warning system we have in Malawi has been funded by donors…. Already, we are seeing sustainability challenges, where the government is unable to provide resources for servicing, maintenance, or replacement’.

Participants from multiple districts cited equipment failure, vandalism, and internet outages as causes of system breakdowns. Some reported receiving no alerts despite forecasts, often due to communication lapses, especially in areas lacking strong institutional support or fallback mechanisms.

Limited capacity for system repair and oversight was another recurring issue. A district official noted that nearly half of the installed systems faced data transmission problems, exposing the risks of over‐reliance on high‐tech forecasting in low‐resource contexts. While valued for their predictive accuracy, scientific systems were thus viewed as vulnerable to institutional and financial constraints, raising questions about their long‐term resilience.

### 3.7 Summary of emergent themes across study sites

The preceding subsections have presented detailed narratives on thematic areas emerging from the field data. To enhance clarity and support comparative analysis, Table 2 summarises the most frequently reported themes, based on frequency across study sites and triangulated participant narratives.

**TABLE 2:** Emergent themes across study sites.

Thematic area	Frequency observed (FGDs/sites)	Illustrative insights
Erosion of IK	Seven out of eight GVHs	Linked to youth disengagement, elder displacement, and ritual decline
Co‐existence and fragmentation	Five out of eight GVHs	Selective blending of scientific and traditional knowledge
Religious and generational tensions	Six out of eight GVHs	Pentecostal teachings reject IK; youth view it as backward
Tokenistic inclusion in policy frameworks	All four disaster‐prone districts and national KIIs	IK is acknowledged rhetorically but rarely budgeted for or enforced

**Source:** author.

These themes serve as anchors for the deeper interpretive analysis presented in the following section.

## 4 DISCUSSION

### 4.1 Epistemic tensions in legitimising disaster knowledge

The declining use of IK in early warning systems in Malawi is not merely a reflection of changing environmental conditions or community preferences; rather, it reflects deeper epistemic tensions over how knowledge is legitimised within disaster governance. As Fricker (2007) argues, epistemic injustice arises when certain knowledge systems are systematically excluded from institutional decision‐making processes, not because they are inherently flawed, but because they do not align with dominant notions of credibility, objectivity, or measurability. The privileging of scientific forecasts over IK in Malawi follows this pattern, with formal disaster institutions implicitly framing knowledge legitimacy using a technocratic lens. This dynamic has been widely documented in disaster studies, where the formalisation of risk governance often privileges scientific epistemologies at the expense of local knowledge systems (Shaw, Sharma, and Takeuchi, 2009; Mercer et al., 2009, 2010; Kelman, Mercer, and Gaillard, 2012).

Evidence from this study supports the claim. Community members frequently reported that their traditional indicators, such as the behaviour of birds, the flowering of trees, or the direction of wind, were dismissed by younger generations and officials alike as ‘*zamakolo*’. As one elderly man in Chikwawa put it: ‘Nowadays, if you say the rains will come because the mangoes are flowering, they just laugh at you. They want to hear it on the radio’. This perception reflects not only generational shifts but also a broader discursive devaluation of IK that parallels global patterns of epistemic exclusion (Jasanoff, 2003, 2004; Fricker, 2007).

The erosion of IK legitimacy is further reinforced by the structure of formal early warning systems, which are designed to favour standardised, quantifiable, and externally verifiable indicators. Studies from other African and Asian contexts have similarly found that such systems are poorly suited to capturing the contextual and experiential nature of IK (Iloka, 2016; Mistry and Berardi, 2016; Trogrlić et al., 2019). Scientific systems in Malawi, often donor‐funded and based on rainfall thresholds, satellite data, or probabilistic forecasting, are considered more reliable by institutions precisely because they conform to these global metrics of validity. Yet, these systems are not infallible: they often face sustainability challenges, delays in dissemination, and limited community uptake. Despite these weaknesses, the institutional bias towards science as authority persists, crowding out other epistemologies that may be more context‐specific, accessible, and socially resonant.

The study findings thus reveal an epistemic paradox: IK is devalued even as its potential contributions, particularly in areas where scientific coverage is limited, are most needed. Similar conclusions have been drawn in recent comparative research that documents how IK can fill spatial and institutional gaps in early warning coverage, especially in marginalised or remote communities (Iticha and Husen, 2019; Cuaton and Su, 2020). This aligns with Jasanoff's (2003) concept of ‘technologies of hubris’, in which formal science is assumed to be superior and self‐sufficient, despite empirical evidence of its limitations in uncertain and resource‐constrained settings. A co‐productionist lens offers an alternative: instead of treating scientific and indigenous systems as competing or hierarchically ordered, it recognises that both are socially constructed and shaped by institutional values, power relations, and histories of knowledge production.

Reframing disaster knowledge in Malawi in this manner calls for a shift away from instrumental debates about which system is ‘better’, towards critical reflection on how legitimacy is defined, by whom, and to what end. This entails not only acknowledging the empirical value of IK but also interrogating the epistemic infrastructures that render it marginal in the first place.

### 4.2 Institutional pathways of marginalisation and symbolic inclusion

While IK continues to be referenced in national disaster policy frameworks and NGO programming in Malawi, its institutional inclusion remains largely symbolic. As the study findings reveal, references to IK in disaster preparedness strategies are frequently superficial, and invoked rhetorically to signal community participation or cultural sensitivity, but rarely accompanied by operational mechanisms for validation, funding, or integration. This pattern reflects Fricker's (2007) concept of epistemic injustice: situations in which individuals or groups are wronged in their capacity as knowers. In the case of IK, this often occurs not through overt exclusion but through superficial inclusion that fails to alter dominant institutional logics.

Interviewees noted that government and NGO‐led interventions regularly acknowledged traditional indicators during project inception or consultation phases but failed to follow up on this during implementation. As one district officer underscored: ‘We talk about IK during community entry, but when it comes to planning, we focus on the scientific data from the Department of Climate Change and Meteorological Services’. This mirrors findings from Chikwawa District, where IK is routinely referenced in consultations but rarely integrated into formal DRR activities or early warning systems (Mwalwimba, Manda, and Ngongondo, 2024). This reveals a dual knowledge hierarchy: IK is consulted but not trusted, documented but not acted upon.

These dynamics are closely linked to the donor‐driven architecture of DRR in Malawi. Most scientific early warning systems, such as river gauges or rainfall thresholds, are externally financed, often tied to specific reporting templates, logframes, and quantitative metrics. These instruments favour standardised data that can demonstrate impact to donors and align with global indicators. IK, by contrast, is relational, contextual, and not easily captured by quantitative benchmarks (Agrawal, 1995; Mistry, 2009). As a result, institutions often overlook it, not because it lacks value, but because it resists institutional logics of audit, attribution, and scalability (Leach, Scoones, and Wynne, 2005).

This marginalisation is further entrenched by the institutional silos between traditional authorities and formal governance structures. In the districts studied, traditional leaders were identified as key custodians of IK, yet their involvement in DRR decision‐making was limited to ceremonial or reactive roles. The broader governance architecture, which is structured around ministerial mandates, climate science inputs, and donor‐financed tools, provides few entry points for sustained use of community‐generated knowledge. As Jasanoff (2004) emphasises in her co‐production framework, knowledge systems are embedded in, and constitutive of, institutional practices. Without institutional transformation, the inclusion of IK is likely to remain tokenistic.

Moreover, when IK is documented, typically in NGO field reports or policy annexes, it is often stripped of its sociocultural context. As several respondents pointed out: ‘You can write about these things, but they will not mean anything unless you see how they work in practice’. This detachment reflects a deeper ontological tension: IK is performative, embodied, and experiential, while institutions seek to render it legible through static documentation (Agrawal, 1995; Mistry, 2009). Such documentation, while useful, risks transforming dynamic knowledge into inert data, which is easily dismissed or instrumentalised.

Consequently, the pathway from symbolic inclusion to meaningful institutionalisation requires more than project‐level interventions. It demands deliberate policy shifts to embed IK in early warning protocols, resource allocation frameworks, and monitoring systems. Without such systemic changes, current engagement with IK risks becoming a performance of inclusion, visible but substantively empty.

### 4.3 Community disengagement, youth, and religious norms as epistemic forces

While institutional structures play a critical role in the marginalisation of IK, the erosion of its legitimacy is also being driven by transformations within communities themselves. The study findings highlight a growing detachment among youth, the shifting influence of religious norms, and intra‐community contradictions that collectively contribute to the unravelling of IK systems from within. These internal dynamics illustrate that epistemic marginalisation is not solely a top‐down phenomenon, but also is embedded in the micro politics of daily life, generational change, and cultural negotiation.

One of the most striking observations from the field was the hesitancy among elders—once the primary custodians of IK—to pass on their knowledge. Respondents frequently mentioned that youth perceive IK as ‘*zamakolo*’ (ancestral or outdated) or even laughable. This shift is not merely a generational gap, but also an epistemic fracture. As Fricker (2012, 2013) argues, knowledge legitimacy is not just a matter of content but also of authority: who is seen as a credible knowledge‐holder and under what conditions. In this case, age and tradition no longer guarantee epistemic authority. Instead, knowledge linked to technology, formal education, or external sources increasingly holds sway.

The reasons for youth disengagement are complex. Rising literacy, digital access, and exposure to global media have reshaped what is seen as ‘valid’ or ‘modern’ knowledge. Scientific forecasts delivered via radio, WhatsApp, or SMS (Short Message Service) are perceived as more accurate and trustworthy, even when such systems are patchy or unreliable. This mirrors the knowledge hierarchies described by Jasanoff (2003), wherein ‘technologies of hubris’—those claiming precision and scientific certainty—displace ‘technologies of humility’—those embedded in local context and social memory. Similar generational shifts are documented by Trogrlić et al. (2019) and Phalira et al. (2022), who observe that younger Malawians increasingly associate credibility with digital or institutional sources rather than ancestral knowledge.

Religion also emerged as a powerful epistemic force. Christianisation in many rural areas has redefined traditional rituals, with some religious leaders actively discouraging the use or even discussion of IK, labelling it as pagan or spiritually dangerous. Practices such as rainmaking ceremonies or interpretations of animal behaviour have been delegitimised within the moral and theological frameworks of modern Christianity. Leach, Scoones, and Wynne (2005) highlight the contested nature of knowledge in governance, where multiple systems vie for authority within shared decision‐making spaces. In the context of disaster knowledge, such epistemic contestation extends beyond science versus IK to include tensions between IK and emergent religious or cultural epistemologies. Importantly, this is not merely a conflict between science and IK, but also between IK and newer forms of cultural power. Dekens (2007) and Mutasa (2015) similarly trace the erosion of traditional ecological knowledge to Christian moral reform and the enduring influence of colonial legacies in DRR.

Even within communities, knowledge systems are contested. Participants cited conflicting interpretations of the same environmental indicators, such as whether a particular type of wind signifies early or late rains. These contradictions weaken the perceived reliability of IK and further reduce its traction, especially among younger or more formally educated community members. In epistemological terms, such intra‐community variation undermines the social cohesion needed for knowledge validation and transmission (Kelman, Mercer, and Gaillard, 2012; Cuaton and Su, 2020).

These findings complicate the notion of ‘community‐based knowledge’ as a homogenous or stable category. Instead, they point to what Jasanoff (2003, 2004) might call fragmented co‐production: a condition where different forms of knowledge co‐exist, conflict, and evolve within overlapping fields of social, cultural, and institutional influence. IK is not disappearing solely because of external suppression; it is also being selectively devalued or displaced from within, through shifting cultural aspirations and everyday epistemic choices.

Ultimately, if IK is to play a meaningful role in disaster preparedness, efforts must go beyond documentation and institutional recognition. They must address the underlying epistemic dynamics within communities themselves: how authority is negotiated, how knowledge is transmitted, and how new norms shape what is remembered, practised, or forgotten.

### 4.4 Beyond integration: toward knowledge pluralism in DRR

Efforts to integrate IK into DRR often rely on a technocratic framing, treating knowledge systems as toolkits to be harmonised or cross‐validated. The findings of this study suggest, however, that such approaches risk oversimplifying complex epistemic realities. As seen in Malawi, the challenges facing IK are not merely technical, such as declining reliability, but also deeply structural, involving questions of legitimacy, authority, and power. This points to the need for a more radical reframing: moving from integration to knowledge pluralism.

Knowledge pluralism, as articulated by Jasanoff (2004) and Leach, Scoones, and Wynne (2005), is not about blending knowledge systems into a single coherent framework; instead, it requires recognising the distinct ontologies, values, and institutional contexts within which different knowledge systems operate. In other words, IK and scientific forecasting are not interchangeable or reducible to a common metric of accuracy. They are produced, legitimised, and deployed in fundamentally different ways, with different assumptions about risk, causality, and appropriate response.

In Malawi, attempts at ‘integration’ often manifest as symbolic inclusion: IK is mentioned in strategy documents or workshops but sidelined in operational plans and funding flows. This resonates with what Jasanoff (2005) refers to as civic epistemologies: the culturally‐specific ways in which societies validate and institutionalise knowledge. In Malawi's disaster governance, civic epistemologies continue to favour scientific over indigenous systems, especially under donor influence, creating hierarchies that are difficult to dismantle. As one development partner remarked: ‘No donor will give you funds because people are seeing ants in this area’. Such views reveal how dominant funding architectures create structural incentives that reinforce epistemic exclusion. This pattern is echoed by Mwalwimba, Manda, and Ngongondo (2024), who observe that although communities in Chikwawa actively apply IK to anticipate seasonal and flood‐related hazards, district‐level DRR actors rarely provide institutional recognition, resources, or formal mechanisms to support its integration.

To move beyond tokenism, DRR strategies must create institutional spaces where multiple knowledge systems can coexist without being forced into equivalence. This requires more than technical adjustments. It calls for shifting the terms of engagement: who gets to define what counts as knowledge, who is involved in interpretation and decision‐making, and what institutional arrangements allow different systems to thrive in parallel.

This also entails recognising the risks of epistemic extractivism, where IK is appropriated for its instrumental value, such as to improve forecasts, without respecting the cultural, relational, or spiritual systems that sustain it (Alcoff, 2022). Such practices reflect Fricker's (2007) epistemic injustice, where individuals or groups are wronged in their capacity as knowers, often through credibility deficits or testimonial exclusion—dynamics that parallel the marginalisation of IK within disaster governance. These concerns resonate with earlier critiques by Agrawal (1995) and Mistry (2009), who caution against isolating knowledge from its sociocultural foundations. Protecting IK, therefore, means protecting the contexts and communities in which it is embedded.

Crucially, this study suggests that IK's value may lie not only in its predictive function but also in its capacity to foster localised forms of resilience, through practices, rituals, and narratives that sustain social cohesion and memory. These are qualities that scientific systems often lack, especially in settings where infrastructure is weak and trust in institutions is fragile. Recognising this broader role of IK requires a paradigm shift: from integrating knowledge systems to enabling dialogue between them, with mutual respect for their distinct logics and limitations. Such perspectives are echoed in the work of Mutasa (2015) and Cuaton and Su (2020), who highlight how rituals and practices linked to IK sustain cohesion and memory during crises.

In practical terms, this means supporting community‐led monitoring systems, creating hybrid training curricula for extension workers, enabling youth engagement platforms that bridge traditional and modern knowledge, and embedding pluralist principles in national DRR policies. It also means rethinking donor criteria, funding mechanisms, and accountability frameworks to accommodate diverse epistemologies.

Only through such pluralistic and reflexive approaches can disaster preparedness systems in Malawi and similar places become more inclusive, sustainable, and attuned to the lived realities of those they aim to serve.

## 5 STUDY LIMITATIONS

While this study offers important insights into the role of IK in disaster early warning systems, several limitations should be acknowledged. The findings are context‐specific and shaped by temporal, environmental, and sociocultural factors that may limit their transferability. The use of purposive sampling, although appropriate for qualitative research, may also introduce selection bias. To address this, efforts were made to ensure diversity and triangulate data across multiple sources. Future research could explore the evolution of IK through longitudinal or comparative designs to strengthen broader applicability.

## 6 CONCLUSION

This study has shown that the declining use of IK in Malawi's disaster early warning systems reflects deeper epistemic tensions rather than mere shifts in hazard exposure or community preferences. While IK continues to offer valuable insights, especially in under‐resourced settings, it remains marginalised by institutional logics that privilege scientific authority, standardised metrics, and donor‐driven priorities.

Rather than calling for simple integration, the findings point to the need for knowledge pluralism: an approach that respects the distinct epistemologies, values, and functions of both scientific and indigenous systems. Reframing DRR in this way requires not only technical solutions, but also political and institutional changes that challenge existing hierarchies of legitimacy.

By foregrounding issues of epistemic injustice and extractivism, this study contributes to broader debates on disaster governance and knowledge politics in climate‐vulnerable contexts. Future research should examine how pluralistic systems can be operationalised—through hybrid platforms, institutional reforms, and participatory governance—to ensure that IK is not merely preserved, but also meaningfully engaged.

Ultimately, sustainable disaster preparedness will depend not on choosing between knowledge systems, but on creating space for them to coexist, complement one another, and co‐produce resilience in diverse and locally relevant ways.

## CONFLICT OF INTEREST STATEMENT

I declare that there are no conflicts of interest—financial, professional, or personal—that could have influenced the research, analysis, or writing of this manuscript.

## FUNDING

This study received no specific funding. However, part of the data used in the paper comes from a study documenting IK funded by Trócaire Malawi, whose support is sincerely acknowledged.

## Data Availability

The data that support the findings of this study are available on request from the corresponding author. The data are not publicly available due to privacy or ethical restrictions.
